# A Structural Modelling Study on Marine Sediments Toxicity

**DOI:** 10.3390/md20080017

**Published:** 2008-06-26

**Authors:** Lorentz Jäntschi, Sorana D. Bolboacã

**Affiliations:** 1 Technical University of Cluj-Napoca, 103-105 Muncii Bvd, 400641 Cluj-Napoca, Romania E-mail: lori@academicdirect.org; 2 Iuliu Hatieganu University of Medicine and Pharmacy Cluj-Napoca, Department of Medical Informatics and Biostatistics, 6 Louis Pasteur, 400349 Cluj-Napoca, Romania

**Keywords:** Toxicity, Ordnance compounds, Molecular Descriptors Family (MDF), Structure-Activity Relationship (SAR), Regression analysis

## Abstract

Quantitative structure-activity relationship models were obtained by applying the Molecular Descriptor Family approach to eight ordnance compounds with different toxicity on five marine species (*arbacia punctulata*, *dinophilus gyrociliatus*, *sciaenops ocellatus*, *opossum shrimp*, and *ulva fasciata*). The selection of the best among molecular descriptors generated and calculated from the ordnance compounds structures lead to accurate monovariate models. The resulting models obtained for six endpoints proved to be accurate in estimation (the squared correlation coefficient varied from 0.8186 to 0.9997) and prediction (the correlation coefficient obtained in leave-one-out analysis varied from 0.7263 to 0.9984).

## 1. Introduction

The effects of marine environment sediment contamination with ordnance compounds received a special attention [1–3]. A number of researches have been conducted near several naval facilities in Puget Sound, WA, revealing that the studied ordnance compounds were not a case for environmental concern in marine sediments [4,5]. The literature also reported that some marine macro algae species (e.g. green alga *acrosiphonia coalita*, red alga *porphyra zezoensis*, and red alga *portieria hornemannii*) have an active role in removal of ordnance compounds [6–8].

**Figure 1:**
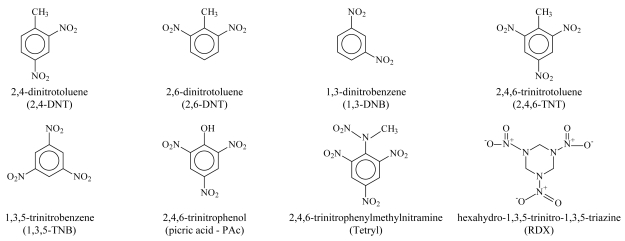
2D structure of ordnance compounds.

The marine sediment toxicity was previously studied by Carr and Nipper [4] for eight ordnance compounds (see Figure 1): 2,4-dinitrotoluene (2,4-DNT), 2,6-dinitrotoluene (2,6-DNT), 1,3-dinitrobenzene (1,3-DNB), 2,4,6-trinitrotoluene (2,4,6-TNT), 1,3,5-trinitrobenzene (1,3,5-TNB), 2,4,6-trinitrophenylmethylnitramine (tetryl), 2,4,6-trinitrophenol (picric acid), and hexahydro-1,3,5-trinitro-1,3,5-triazine (Royal Demolition Explosive - RDX). The reproduction of the *polychaete* and the embryological development of *arbacia punctulata* have been identified as most sensitive species and endpoints [4] while tetryl and 1,3,5-trinitrobenzen are considered as the most toxic ordnance compounds [4].

The main objective of the present research was to identify and to quantify the relationship between the structure of eight ordnance compounds and their marine toxicity by using the Molecular Descriptors Family on the Structure-Activity Relationships approach.

## 2. Material and Method

### 2.1. Ordnance compounds and associated toxicities

The experimental toxicities of eight ordnance compounds on *arbacia punctulata* (sea urchin), *dinophilus gyrociliatus* (polychaete), *sciaenops ocellatus* (redfish), *opossum shrimp* (mysid), and *ulva fasciata* (macro-alga) were taken from a previously reported research [4]. The toxicity on nine endpoints was analyzed. The toxicities were expressed as [9]:

- Effective Concentration to 50% of the organism (EC_50_), defined as the effective concentration of toxin in aqueous solution that produces a specific measurable effect in 50% of the test organisms within the stated study time (see Table 1).
- No Observed Effect Concentration (NOEC) defined as the highest concentration of toxicant to which organisms are exposed in a full or partial life-cycle test, that determine no observable adverse effects on the test organisms (the highest concentration of toxicant in which the values for the observed responses are not statistically different from the controls) (see Table 2).
- Lowest Observed Effect Concentration (LOEC) defined as the lowest concentration of toxicant to which organisms are exposed in a full or partial life-cycle test, which causes adverse effects on the test organisms (where the values for the observed responses are statistically significant different from the controls) (see Table 3).

The experimental data (expressed as mg/L) were transformed in logarithmic scale and are presented in Table 1 for EC_50_, Table 2 for NOEC, and Table 3 for LOEC.

### 2.2. Modelling procedure

The toxicities of the ordnance compounds on the investigated marine species were modelled by using the molecular descriptors family on the structure-activity relationships (MDF SARs) [10]. The MDF SARs approach proved its estimated ability and predictive power on classes of compounds with different activity or property [11–19]. The steps applied in molecular modelling were as follows [10]:

- Step 1: Bi- and tri-dimensional representation of the investigated ordnance compounds. This task was done by using a molecular modelling software, HyperChem;
- Step 2: Preparation of the compounds for modelling, optimization of geometry and creation of the file with experimental data;
- Step 3: Construction, generation, calculation and filtration of the molecular descriptors family. The information extracted from the compound’s structure was used in order to construct, generate, and calculate the molecular descriptors. The obtained descriptors were stored into a database. A biases algorithm was applied in order to delete identically recordings. Seven characteristics were considered in the construction of descriptors: Compound geometry or topology (the 7^th^ letter in the descriptor name); Atomic property (e.g. atomic relative mass, atomic partial charge, cardinality, atomic electro negativity, group electro negativity, number of directly bonded hydrogen’s – the 6^th^ letter); Interaction descriptor (the 5^th^ letter); Overlapping interaction models (the 4^th^ letter); Molecular fragmentation criterion (the 3^rd^ letter) [20,21]; Cumulative method of properties fragmentation (the 2^nd^ letter); and Linearization procedure applied in molecular descriptor generation (the 1^st^ character).
- Step 4: Search and identification of the most significant MDF SAR models with one molecular descriptor. The following criteria were used: squared correlation coefficient, standard error of estimated, statistical parameters of the regression model.
- Step 5: Validation of the obtained models. A leave-one-out cross-validation analysis was performed. The cross-validation leave-one-out score, standard error of predict and Fisher parameter were calculated and interpreted [19].
- Step 6: The analysis of the models. The stability of the model (the lowest the difference between squared correlation coefficient and leave-one-out cross-validation score is, the stable de model was considered), and the predictive power was assessed. The toxicity of the ordnance compounds for which the experimental determinations were not available as values (see n.a. from Tables 1 – 3) were predicted based on the obtained models by using online software^2^.

## 3. Results and Discussion

The MDF SAR monovariate models with estimated and predictive abilities on investigated endpoints for studied ordnance compounds were identified and are presented in Table 4 for EC_50_, Table 5 for NOEC, and Table 6 for LOEC.

The analysis of the Tables 4 – 6 revealed that all monovariate regression models are statistically significant at a significance level of 5% (p < 0.0001). Note that significance of the descriptor’s name is explained on Material and Method section, “Step 3” and is explained in the results tables below descriptor names (see the followings: Dominant Atomic Property, Interaction via, Interaction Model, and Structure on Activity Scale).

**Figure 2:**
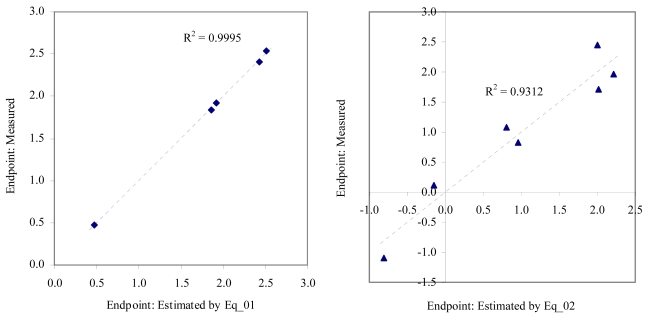
Relationship between experimental and estimated EC_50_: fertilization (Eq_01, left hand graphic), and embryological development of *sea urchin* (Eq_02, right hand graphic).

**Figure 3:**
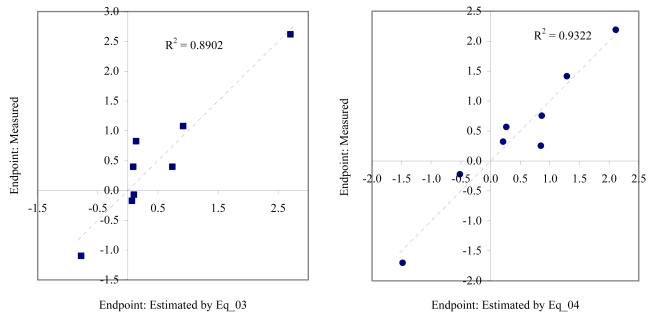
Relationship between experimental and estimated EC_50_: germination of *sea urchin* (Eq_03, left hand graphic), and survival and reproductive success of *polychaete* (Eq_04, right hand graphic).

**Figure 4:**
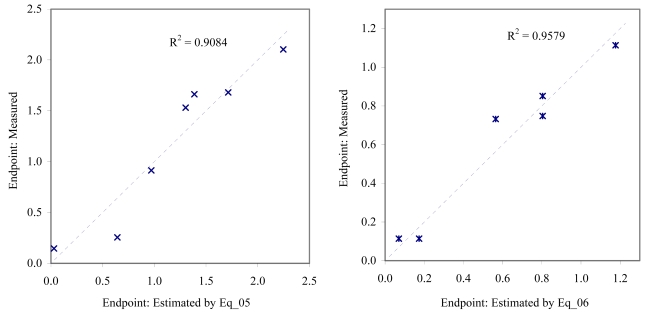
Relationship between experimental and estimated EC_50_: larvae survival of *redfish* (Eq_05, left hand graphic), and juveniles survival of *mysid* (Eq_06, right hand graphic).

**Figure 5:**
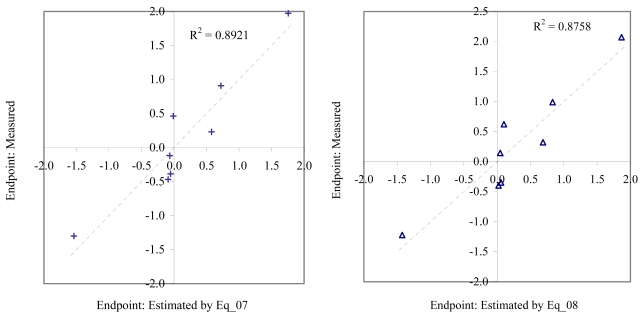
Relationship between experimental and estimated EC_50_: germling length (Eq_07, left hand graphic), and germling cell number of *macro-alga* (Eq_08, right hand graphic).

**Figure 6:**
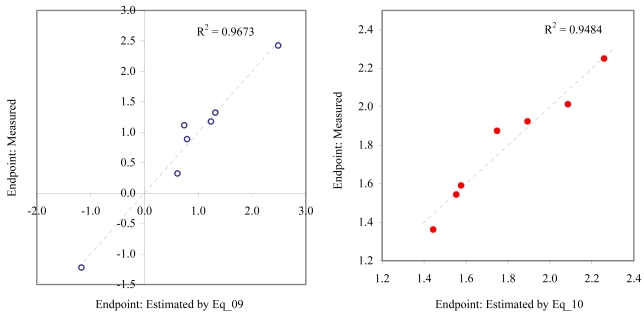
Relationship between experimental and estimated EC_50_: survival of *macro-alga* (Eq_09, left hand graphic), and NOEC as fertilization of *sea urchin* (Eq_10, right hand graphic).

**Figure 7:**
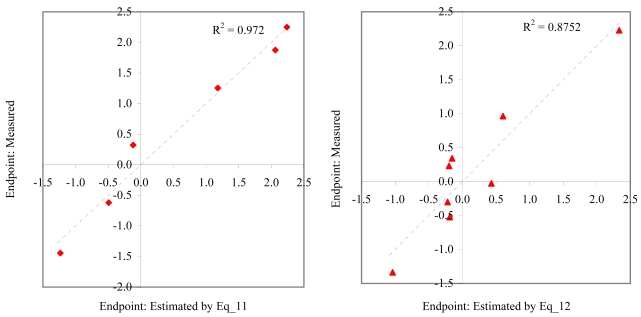
Relationship between experimental and estimated NOEC: embryological development (Eq_11, left hand graphic), and germination of *sea urchin* (Eq_12, right hand graphic).

**Figure 8:**
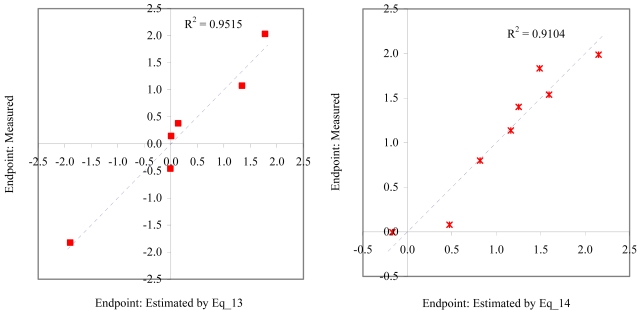
Relationship between experimental and estimated NOEC: laid eggs/female of *polychaete* (Eq_13, left hand graphic), and larvae survival of *redfish* (Eq_14, right hand graphic).

**Figure 9:**
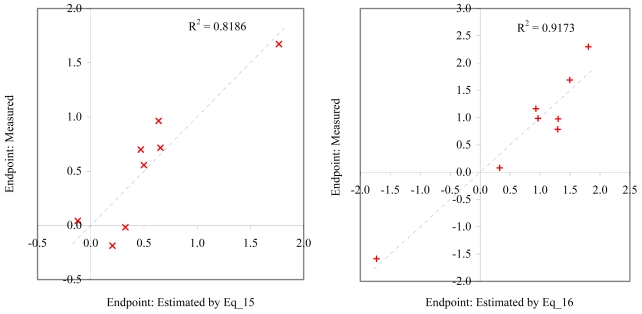
Relationship between experimental and estimated NOEC: survival of *mysid* (Eq_15, left hand graphic), and survival of *macro-alga* (Eq_16, right hand graphic).

**Figure 10:**
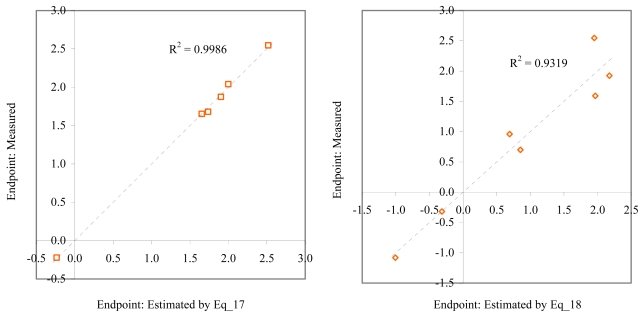
Relationship between experimental and estimated LOEC: fertilization (Eq_17, left hand graphic), and embryological development of *sea urchin* (Eq_18, right hand graphic).

**Figure 11:**
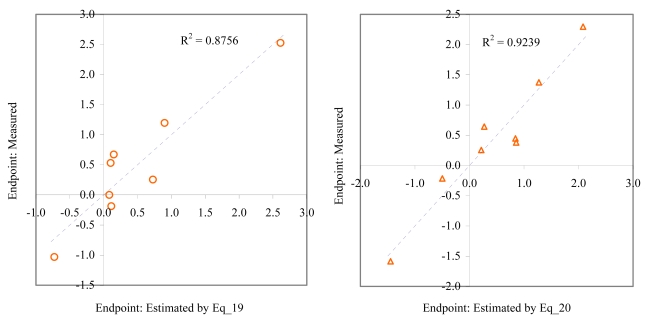
Relationship between experimental and estimated LOEC: germination of *sea urchin* (Eq_19, left hand graphic), and laid eggs/female of *polychaete* (Eq_20, right hand graphic).

**Figure 12:**
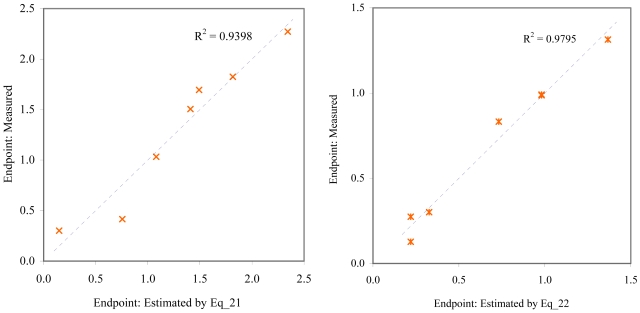
Relationship between experimental and estimated LOEC: larvae survival of *redfish* (Eq_21, left hand graphic), and survival of *mysid* (Eq_22, right hand graphic).

**Figure 13:**
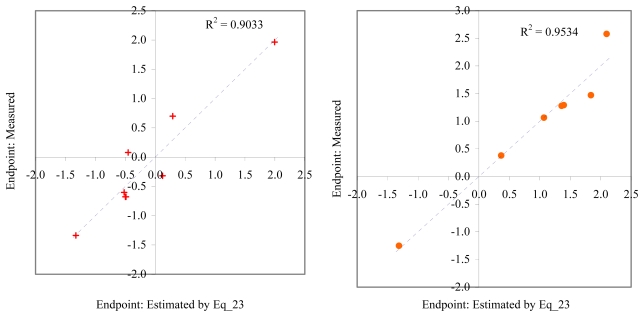
Relationship between experimental and estimated LOEC: germling length and cell number (Eq_22, left hand graphic), and survival of *macro-alga* (Eq_24, right hand graphic).

The goodness-of-fit of all models were close to the highest value (one): greater than 0.93 for EC_50_ (see Table 4) and LOEC (see Table 6), and 0.90 for NOEC (see Table 5). The goodness-of-fit of the models is also sustained by the values of standard error of estimated which never took values greater than 0.42 (see the values of standard error of estimated (s), Tables 4 – 6). The relationship between the investigated toxicity and molecular descriptor used as independent variable was very good (see Figures 2 – 13).

Therefore, more than eighty-one percent of the activity of interest on studied ordnance compounds can be explained by the linear relationship with the variation of molecular descriptors generated strictly based on the information extracted from the ordnance compounds structure (see values of coefficient of determination – R^2^ from Figures 2 – 13). The lowest determination ability was obtained for the juveniles’ survival of mysid (with R^2^ = 0.8186). The highest determination was obtained for fertilization of sea urchin (R^2^ = 0.9995). In seventy-five percent of cases the determination ability was higher than 0.9000.

The stability of each model was investigated in a cross-validation leave-one-out analysis. The values of the cross-validation leave-one-out score sustained the validity of the models. The lowest cross-validation leave-one-out score was of 0.7263. The values where higher than:

- 0.7500 in twenty-three out of twenty-four cases;
- 0.8000 in twenty-two out of twenty-four cases;
- 0.8500 in fifteen out of twenty-four cases;
- 0.9000 in nine out of twenty-four cases.

The lowest value of the cross-validation leave-one-out score was obtained by Eq_15 (see Table 5) being in accordance with the value of the correlation coefficient. The highest cross-validation leave-one-out score was obtained by Eq_01 (see Table 4).

The stability of the obtained models could be expressed by the difference between the determination coefficient and the cross-validation leave-one-out score. The model from Eq_01 obtained the lowest value of 0.0011 while the model from Eq_11 obtained the highest value of 0.0923. The differences between coefficient of determination and leave-one-out cross-validation score did not exceed 0.1, sustaining the absence of over fitted model and/or the absence of outliers. Therefore, it can be concluded that the lowest ability in identification and quantification the relationships between structures of the ordnance compounds and toxicity was obtained for juveniles’ survival of mysid when the NOEC was the investigated toxicity.

The obtained MDF SAR models are valid according with the criteria of Erikson *et al.* [22] (see the statistical parameters of all models presented in Eq_01 – Eq_24, Tables 4 – 6, and Figures 2 – 13).

In the regard of the type of relationships between ordnance compounds structures and associated toxicities on investigated species it can say that:

- The EC_50_ on the investigated endpoints (different species, see Table 4) revealed to be of geometrical nature and directly related with the atomic partial charge (almost 44% of investigated endpoints showed to be of topological nature, see Table 4).
- The NOEC on the investigated endpoints (different species, see Table 5) revealed also to be of geometrical nature and directly related with the partial charge (the topological nature was observed in 3 cases out of seven, while the relationship with compounds electronegativity was observed in 1 case out of 7 cases, see Table 5).
- The LOEC on the investigated endpoints (different species, see Table 6) revealed also to be of geometrical nature (the topological nature was identified in 3 cases out of 8 investigated) and directly related with the partial charge (the relationship with compounds cardinality was observed in 1 case out of 8 investigated, see Table 5).

The activities of ordnance compounds without reliable experimental data (expressed as values greater than a number, see Tables 1 – 3) were predicted by using the obtained models (Tables 4 – 6). The results expressed as the values of the molecular descriptors and predicted activities are presented in Table 7.

The predicted toxicities on different species calculated for studied ordnance compounds need to be validated. This can be done easily once the experimental toxicities are measure. The MDF SAR approach proved to be a useful method in characterization of ordnance compounds toxicities on investigated marine species, offering valid and reliable models. The limited number of the compounds investigated represents the main limitation of the study. The impossibility of validation the predicted toxicities (see Table 7) is another limitation of the study. The obtained MDF SARs models were obtained on small samples, thus further investigations must be done for the validation of the approach.

## Conclusion

The MDF SAR approach proved its usefulness in characterization of the toxicity of ordnance compounds. The relationship between ordnance compounds structure and their toxicities revealed to be in the majority of the cases of geometrical nature and directly related with the partial charge for all three types of investigated toxicities.

## Figures and Tables

**Table 1:** Ordnance compounds toxicity: experimental EC_50_.

Specie	Endpoint	2,4-DNT	2,6-DNT	1,3-DNB	2,4,6-TNT	1,3,5-TNB	PAc	Tetryl	RDX
sea urchin	fertilization	1.8325	n.a.	2.4116	n.a.	1.9243	2.5428	0.4771	n.a.
	embryological development	1.7110	0.8261	1.9638	1.0792	0.1139	2.4487	−1.0969	n.a.
	germination	0.3979	0.8261	−0.0706	0.3979	−1.0969	2.6180	−0.1739	1.0792

polychaete	survival and reproductive success	0.7559	0.3222	0.5682	0.2553	−0.2218	2.1903	−1.6990	1.4150

redfish	larvae survival	1.6812	1.5315	1.6628	0.9138	0.1461	2.1038	0.2553	n.a.

mysid	juveniles survival	0.7324	0.7482	0.8513	−0.0088	0.1139	1.1139	0.1139	1.6628

macro-alga	germling length	0.2304	0.4624	−0.3872	−0.1192	−1.3010	1.9731	−0.4685	0.9085
	germling cell number	0.3222	0.6232	−0.3468	0.1461	−1.2218	2.0719	−0.3979	0.9912
	survival	1.3222	1.1139	1.1761	0.8865	0.3222	2.4232	−1.2218	n.a.

EC_50_ = Effective Concentration to 50% of the organism expressed as logarithmic scale;

2,4-DNT = 2,4-dinitrotoluene; 2,6-DNT = 2,6-dinitrotoluene;

1,3-DNB = 1,3-dinitrobenzene; 2,4,6-TNT = 2,4,6-trinitrotoluene;

1,3,5-TNB = 1,3,5-trinitrobenzene; PAc = 2,4,6-trinitrophenol (picric acid);

Tetryl = 2,4,6-trinitrophenylmethylnitramine;

RDX = hexahydro-1,3,5-trinitro-1,3,5-triazine (Royal Demolition Explosive); n.a. = not available (experimental data expressed as greater than – mg/L)

**Table 2:** Ordnance compounds toxicity: experimental NOEC values.

Specie	Endpoint	2,4-DNT	2,6-DNT	1,3-DNB	2,4,6-TNT	1,3,5-TNB	PAc	Tetryl	RDX
sea urchin	fertilization	1.5911	1.3617	1.9243	2.0128	1.5441	2.2504	n.a.	1.8751
	embryological development	1.2553	n.a.	n.a.	0.3222	−0.6198	2.2504	−1.4437	1.8751
	germination	−0.0269	0.3424	−0.5229	0.2304	−1.3372	2.2279	−0.3010	0.9638

polychaete	laid eggs/female	n.a.	n.a.	0.3802	0.1461	−0.4559	2.0334	−1.8239	1.0755

redfish	larvae survival	1.5391	1.1367	1.4014	0.7993	−0.0044	1.9868	0.0792	1.8325

mysid	survival	0.5563	0.6990	0.7160	−0.1871	−0.0177	0.9638	0.0414	1.6721

macro-alga	germling length and cell number	n.a.	n.a.	n.a.	n.a.	−1.5376	n.a.	−1.0088	n.a.
	survival	0.9777	1.1644	0.9868	0.7853	0.0792	2.2989	−1.5850	1.6902

NOEC = No Observed Effect Concentration;

2,4-DNT = 2,4-dinitrotoluene; 2,6-DNT = 2,6-dinitrotoluene;

1,3-DNB = 1,3-dinitrobenzene; 2,4,6-TNT = 2,4,6-trinitrotoluene;

1,3,5-TNB = 1,3,5-trinitrobenzene; PAc = 2,4,6-trinitrophenol (picric acid);

Tetryl = 2,4,6-trinitrophenylmethylnitramine; RDX = hexahydro-1,3,5-trinitro-1,3,5-triazine (Royal Demolition Explosive);

n.a. = not available (experimental data expressed as greater than a value – mg/L)

**Table 3:** Ordnance compounds toxicity: experimental LOEC values.

Specie	Endpoint	2,4-DNT	2,6-DNT	1,3-DNB	2,4,6-TNT	1,3,5-TNB	PAc	Tetryl	RDX
sea urchin	fertilization	1.8751	1.6532	2.0414	n.a.	1.6812	2.5465	−0.2218	n.a.
	embryological development	1.5911	0.6990	1.9243	0.9590	−0.3188	2.5465	−1.0809	n.a.
	germination	0.2553	0.6721	−0.1871	0.5315	−1.0315	2.5263	0.0000	1.1959

polychaete	laid eggs/female	0.3802	0.2553	0.6435	0.4472	−0.2147	2.2967	−1.5850	1.3747

redfish	larvae survival	1.8248	1.5051	1.6955	1.0334	0.3010	2.2718	0.4150	n.a.

mysid	survival	0.8325	0.9912	0.9868	0.1271	0.2742	1.3139	0.3010	n.a.

macro-alga	germling length and number	cell −0.3188	0.0792	−0.6778	−0.6778	−1.3372	1.9638	−0.6021	0.6990

	survival	1.2788	1.4713	1.2923	1.0645	0.3802	2.5786	−1.2518	n.a.

LOEC = Lowest Observed Effect Concentration;

2,4-DNT = 2,4-dinitrotoluene; 2,6-DNT = 2,6-dinitrotoluene;

1,3-DNB = 1,3-dinitrobenzene; 2,4,6-TNT = 2,4,6-trinitrotoluene;

1,3,5-TNB = 1,3,5-trinitrobenzene; PAc = 2,4,6-trinitrophenol (picric acid);

Tetryl = 2,4,6-trinitrophenylmethylnitramine; RDX = hexahydro-1,3,5-trinitro-1,3,5-triazine (Royal Demolition Explosive)

n.a. = not available (experimental data expressed as greater than a value – mg/L)

**Table 4:** MDF SAR monovariate models: EC_50_.

	sea urchin		
Endpoint	fertilization	embryological development	germination
MDF SAR Equation	Ŷ = − 0.16 – 0.37·X	Ŷ = −7.09 – 1.09·X	Ŷ = −1.50 + 6.28·10^−2^·X
(Eq_no)	Eq_01	Eq_02	Eq_03
Correlation coefficient (r)	0.9997	0.9650	0.9435
95% confidence interval for r	[0.9885–0.9999]	[0.6193–0.9973]	[0.5477–0.9942]
Standard error of estimated (s)	0.02	0.35	0.39
Fisher parameter (p-value)	5674 (p = 5.16·10^−6^)	68 (p = 4.32·10^−4^)	49 (p = 4.32·10^−4^)
Cross-validation leave-one-out score (r_cv-loo_^2^)	0.9984	0.8460	0.8333
Sample size	5	7	8
Descriptor (X)	LIMmwQt	lNPmfQt	aIDmjQg
Dominant Atomic Property	Partial charge (**Q**)	Partial charge (**Q**)	Partial charge (**Q**)
• Interaction via	Bonds (**t**opology)	Bonds (**t**opology)	Space (**g**eometry)
• Interaction Model	Q^2^/d	Q^2^/d^2^	(Q·d)^−1^
• Structure on Activity Scale	Logarithmic	Logarithmic	Inversed
***Endpoint***	***survival and reproductive success (polychaete)***	***larvae survival (redfish)***	***juveniles survival (mysid)***

MDF SAR Equation	Ŷ = −1.73 + 16.91·X	Ŷ = 0.28 − 1.31·X	Ŷ = 3.93 − 0.80·X
Eq	Eq_04	Eq_05	Eq_06
Correlation coefficient (r)	0.9655	0.9531	0.9787
95% confidence interval	[0.7000–0.9965]	[0.5186–0.9963]	[0.7511–0.9983]
Standard error of estimated (s)	0.32	0.25	0.10
Fisher parameter (p-value)	82 (p = 1.00·10^−4^)	50 (p = 8.92·10^−4^)	114 (p = 1.25·10^−4^)
Cross-validation leave-one-out score (r_cv-loo_^2^)	0.8852	0.8412	0.9267
Sample size	8	7	7
MDF Descriptor	anDRJQt	LHDmjQg	imMrtCg
Dominant Atomic Property	Partial charge (**Q**)	Partial charge (**Q**)	Cardinality (**C**)
• Interaction via	Bonds (**t**opology)	Space (**g**eometry)	Space (**g**eometry)
• Interaction Model	Q·d	(Q·d)^−1^	C^2^/d^4^
• Structure on Activity Scale	Inversed	Logarithmic	Inversed
	***macro-alga***		
***Endpoint***	***germling length***	***germling cell number***	***gurvival***

MDF SAR Equation	Ŷ = −6.13 − 1.88·X	Ŷ = −6.02 − 1.87·X	Ŷ = −0.79 − 102.72·X
Eq	Eq_07	Eq_08	Eq_09
Correlation coefficient (r)	0.9445	0.9359	0.9835
95% confidence interval	[0.7170–0.9901]	[0.6790–0.9885]	[0.8884–0.9976]
Standard error of estimated (s)	0.35	0.38	0.22
Fisher parameter (p-value)	50 (p = 4.09·10^−4^)	42 (p = 6.28·10^−4^)	148 (p = 6.65·10^−5^)
Cross-validation leave-one-out score (r_cv-loo_^2^)	0.8045	0.7933	0.9503
Sample size	8	8	7
Descriptor (X)	LIDmjQg	LIDmjQg	IAPmtQt
Dominant Atomic Property	Partial charge (**Q**)	Partial charge (**Q**)	Partial charge (**Q**)
• Interaction via	Space (**g**eometry)	Space (**g**eometry)	Bonds (**t**opology)
• Interaction Model	(Q·d) ^−1^	(Q·d) ^−1^	Q^2^·d^−4^
• Structure on Activity Scale	Logarithm	Logarithm	Identity

d = distance

**Table 5:** MDF SAR monovariate models: NOEC.

	sea urchin		
Endpoint	fertilization	embryological development	germination
MDF SAR Equation	Ŷ = 1.42 + 0.17·X	Ŷ = −1.27 + 1.27·10^−3^·X	Ŷ = −1.74 + 6.08·10^−2^·X
(Eq_no)	Eq_10	Eq_11	Eq_12
Correlation coefficient (r)	0.9739	0.9859	0.9355
95% confidence interval for r	[0.8283–0.9962]	[0.8721–0.9985]	[0.6772–0.9885]
Standard error of estimated (s)	0.08	0.27	0.41
Fisher parameter (p-value)	92 (p = 2.09·10^−4^)	139 (p = 2.97·10^−4^)	42 (p = 6.38·10^−4^)
Cross-validation leave-one-out score (r_cv-loo_^2^)	0.9101	0.9417	0.8105
Sample size	7	6	8
Descriptor (X)	ASPmwQg	asmrfQt	aIDmjQg
Dominant Atomic Property	Partial charge (**Q**)	Partial charge (**Q**)	Partial charge (**Q**)
• Interaction via	Space (**g**eometry)	Bonds (**t**opology)	Space (**g**eometry)
• Interaction Model	Q^2^·d^−1^	Q^2^·d^−2^	(Q·d) ^−1^
• Structure on Activity Scale	Absolute	Inversed	Inversed
***Endpoint***	***survival and reproductive success (polychaete)***	***larvae survival (redfish)***	***juveniles survival (mysid)***

MDF SAR Equation	Ŷ = −10.25 − 1.42·X	Ŷ = 9.35·10^−2^ − 1.37·X	Ŷ = 19.24 + 668.36·X
Eq	Eq_13	Eq_14	Eq_15
Correlation coefficient (r)	0.9754	0.9542	0.9048
95% confidence interval	[0.7861–0.9974]	[0.7616–0.9919]	[0.5521–0.9828]
Standard error of estimated (s)	0.32	0.24	0.28
Fisher parameter (p-value)	78 (p = 8.98·10^−4^)	61 (p = 2.33·10^−4^)	27 (p = 2.01·10^−3^)
Cross-validation leave-one-out score (r_cv-loo_^2^)	0.9060	0.8394	0.7263
Sample size	6	8	8
MDF Descriptor	LsmrfQg	LHDmjQg	iBPMwEt
Dominant Atomic Property	Partial charge (**Q**)	Partial charge (**Q**)	Electronegativity (**E**)
• Interaction via	Space (**g**eometry)	Space (**g**eometry)	Bonds (**t**opology)
• Interaction Model	Q^2^·d^−2^	Q^2^·d^−2^	E^2^·d^−1^
• Structure on Activity Scale	Logarithm	Logarithm	Inversed
***Endpoint***	***survival (macro-alga)***		
	
MDF SAR Equation	Ŷ = 3.71 − 1.28·X		
Eq	Eq_16		
Correlation coefficient (r)	0.9578		
95% confidence interval	[0.7786–0.9925]		
Standard error of estimated (s)	0.36		
Fisher parameter (p-value)	67 (p = 1.83·10^−4^)		
Cross-validation leave-one-out score (r_cv-loo_^2^)	0.8532		
Sample size	8		
Descriptor (X)	LnDRJQt		
Dominant Atomic Property	Partial charge (**Q**)		
• Interaction via	Bonds (**t**opology)		
• Interaction Model	Q·d		
• Structure on Activity Scale	Logarithm		

d = distance

**Table 6:** MDF SAR monovariate models: LOEC.

	sea urchin		
Endpoint	fertilization	embryological development	germination
MDF SAR Equation	Ŷ = 0.57 − 47.56·X	Ŷ = −7.62 −1.14·X	Ŷ = −1.43 + 6.02·10^−2^·X
(Eq_no)	Eq_17	Eq_18	Eq_19
Correlation coefficient (r)	0.9993	0.9653	0.9357
95% confidence interval for r	[0.9932–0.9999]	[0.7771–0.9950]	[0.6781–0.9885]
Standard error of estimated (s)	0.04	0.36	0.40
Fisher parameter (p-value)	2781 (p = 7.74·10−^7^)	68 (p = 4.22·10−^4^)	42 (p = 6.33·10−^4^)
Cross-validation leave-one-out score (r_cv-loo_^2^)	0.9962	0.8753	0.8140
Sample size	6	7	8
Descriptor (X)	IAPmfQt	lNPmfQt	aIDmjQg
Dominant Atomic Property	Partial charge (**Q**)	Partial charge (**Q**)	Partial charge (**Q**)
• Interaction via	Bonds (**t**opology)	Bonds (**t**opology)	Space (**g**eometry)
• Interaction Model	Q^2^·d^−2^	Q^2^·d^−2^	Q^2^·d^−2^
• Structure on Activity Scale	Identity	Logarithm	Inversed
***Endpoint***	***survival and reproductive success (polychaete)***	***larvae survival (redfish)***	***juveniles survival (mysid)***

MDF SAR Equation	Ŷ = −1.69 + 16.60·X	Ŷ = 0.39 − 1.30·X	Ŷ = 4.22 − 0.83·X
Eq	Eq_20	Eq_21	Eq_22
Correlation coefficient (r)	0.9612	0.9694	0.9897
95% confidence interval	[0.7949–0.9931]	[0.8012–0.9956]	[0.9290–0.9985]
Standard error of estimated (s)	0.34	0.20	0.07
Fisher parameter (p-value)	73 (p = 1.42·10^−4^)	78 (p = 3.09·10^−4^)	239 (p = 2.06·10^−5^)
Cross-validation leave-one-out score (r_cv-loo_^2^)	0.8763	0.8844	0.9585
Sample size	8	7	7
MDF Descriptor	anDRJQt	LHDmjQg	imMrtCg
Dominant Atomic Property	Partial charge (**Q**)	Partial charge (**Q**)	Cardinality (**C**)
• Interaction via	Bonds (**t**opology)	Space (**g**eometry)	Space (**g**eometry)
• Interaction Model	Q·d	Q^2^·d^−2^	Q^2^·d^−4^
• Structure on Activity Scale	Inversed	Logarithm	Inversed
	***macro-alga***		
***Endpoint***	***germling length and cell number***		***survival***

MDF SAR Equation	Ŷ = −2.02 + 5.99·10^−2^·X		Ŷ = 3.69 + 0.11·X
Eq	Eq_23		Eq_24
Correlation coefficient (r)	0.9504		0.9764
95% confidence interval	[0.7439–0.9912]		[0.8436–0.9966]
Standard error of estimated (s)	0.35		0.28
Fisher parameter (p-value)	56 (p = 2.94·10^−4^)		102 (p = 1.62·10^−4^)
Cross-validation leave-one-out score (r_cv-loo_^2^)	0.8686		0.9091
Sample size	8		7
Descriptor (X)	aIDmjQg		iIDdPQg
Dominant Atomic Property	Partial charge (Q)		Partial charge (Q)
• Interaction via	Space (geometry)		Space (geometry)
• Interaction Model	Q^2^·d^−2^		Q^2^
• Structure on Activity Scale	Inversed		Inversed

d = distance

**Table 7:** Predicted activities of ordnance compounds by using the MDF SAR mono-variate models.

Activity - Specie	Toxicity	Compound	Eq_	X	Ŷ_Pred_
Fertilization - *sea urchin*	EC_50_	2,6-DNT	01	−4.9295	1.6618
	EC_50_	2,4,6-TNT	01	−6.6904	2.3116
	EC_50_	RDX	01	−5.8418	1.9984
	LOEC	RDX	17	−0.0398	2.4593

Embryological development - *sea urchin*	EC_50_	RDX	02	−7.9917	1.6018
	NOEC	2,6-DNT	11	6355.74	6.8112
		1,3-DNB	11	2900.88	2.4159
	LOEC	RDX	18	−5.8418	1.9984

Fertilization - *sea urchin*	NOEC	Tetryl	10	333.40	56.8491

Larvae survival - *redfish*	EC_50_	RDX	05	−1.0141	1.6124
	LOEC	RDX	21	−1.0141	1.7153

Juveniles survival - *mysid*	EC_50_	RDX	06	4.6574	0.1832

Survival - *mysid*	LOEC	RDX	22	4.6574	0.3365

Laid eggs/female - *polychaete*	NOEC	2,4-DNT	13	−7.2544	0.0519
		2,6-DNT	13	−8.5506	1.8932

Survival - *macro-alga*	EC_50_	RDX	09	−0.0562	4.9762
	LOEC	RDX	24	32.7066	−0.1848

X = value of the molecular descriptors used by MDF SAR equation – see Tables 4 – 6;

2,6-DNT = 2,6-dinitrotoluene; 2,4,6-TNT = 2,4,6-trinitrotoluene; RDX = hexahydro-1,3,5-trinitro-1,3,5-triazine;

Ŷ_Pred_ = predicted activity

## Footnotes

2 SARs (2005) Virtual Library of Free Software. Available online; http://l.academicdirect.org/Chemistry/SARs/MDF_SARs/loo/; accessed on 20 October 2007.
